# Achieving equity within universal health coverage: a narrative review of progress and resources for measuring success

**DOI:** 10.1186/s12939-014-0072-8

**Published:** 2014-10-10

**Authors:** Anna M Rodney, Peter S Hill

**Affiliations:** School of Population Health, University of Queensland, Room 116, Level 1, Public Health Building, Herston, Brisbane, QLD 4006 Australia; Global Health Systems, School of Population Health, University of Queensland, Brisbane, Australia

**Keywords:** Universal health coverage, UHC, Universal health care, Equity, Inequity, Sustainable development goals, SDGs

## Abstract

**Introduction:**

Equity should be implicit within universal health coverage (UHC) however, emerging evidence is showing that without adequate focus on measurement of equity, vulnerable populations may continue to receive inadequate or inferior health care. This study undertakes a narrative review which aims to: (i) elucidate how equity is contextualised and measured within UHC, and (ii) describe tools, resources and lessons which will assist decision makers to plan and implement UHC programmes which ensure equity for all.

**Methods:**

A narrative review of peer-reviewed literature published in English between 2005 and 2013, retrieved from PubMed via the search words, ‘universal health coverage/care’ and ‘equity/inequity’ was performed. Websites of key global health organizations were also searched for relevant grey literature. Papers were excluded if they failed to focus on equity (of access, financial risk protection or health outcomes) as well as focusing on one of the following: (i) the impact of UHC programmes, policies or interventions on equity (ii) indicators, measurement, monitoring and/or evaluation of equity within UHC, or (iii) tools or resources to assist with measurement.

**Results:**

Eighteen journal articles consisting mostly of secondary analysis of country data and qualitative case studies in the form of commentaries/reviews, and 13 items of grey literature, consisting largely of reports from working groups and expert meetings focusing on defining, understanding and measuring inequity in UHC (including recent drafts of global/country monitoring frameworks) were included.

**Discussion:**

The literature advocates for progressive universalism addressing monetary and non-monetary barriers to access and strengthening existing health systems. This however relies on countries being effectively able to identify and reach disadvantaged populations and estimate unmet need. Countries should assess the new WHO/WB-proposed framework for its ability to adequately track the progress of disadvantaged populations in terms of achieving equitable access, effective coverage and financial risk protection within their own settings.

**Conclusions:**

Recently published resources contextualise equity as a measurable component of UHC and propose several useful indicators and frameworks. Country case-studies also provide useful lessons and recommendations for planning and implementing equitable UHC which will assist other countries to consider their own requirements for UHC monitoring and evaluation.

**Electronic supplementary material:**

The online version of this article (doi:10.1186/s12939-014-0072-8) contains supplementary material, which is available to authorized users.

## Introduction

Universal health coverage (UHC), defined by the 2005 World Health Assembly as “*access to key promotive, preventive, curative and rehabilitative health interventions for all at an affordable cost, thereby achieving equity in access*”, [1] has become a rallying call for global health. By 2012, over 90 countries had formally endorsed the United Nations Resolution to make UHC a key global health objective [2] and to date, more than 70 countries have requested technical assistance from the World Health Organization (WHO) in implementing UHC [3]. Recently, UHC has been heralded as ‘the third global health transition’ [4] and has featured prominently in discourse around the post-2015 Sustainable Development Goals (SDGs). With health likely to receive only one goal, UHC has been strongly advocated for its ability to provide an umbrella goal, incorporating both the unfinished infectious, maternal and child health (MCH) focus of the millennium development goals (MDGs) as well as the emerging non-communicable disease (NCD) agenda [5].

As the end of the MDGs draws near and discussions converge on the future of global health, now is a critical time to stop and take stock. Failure to address equity was deemed the most serious shortcoming of the MDGs [6] with many countries neglecting the most vulnerable populations by focusing instead on quantified targets which did not, for the most part, promote universal cover [7]. While equitable access and financing should be integral outcomes of UHC, evidence emerging from country case studies is however showing that in spite of gains in health coverage, and/or the overall level of population health, inequities can persist or even widen when there is insufficient focus on equity [8-12]. To ensure that the push towards UHC does not make the same mistakes and leave the same disadvantaged populations behind, countries and development partners must make equity an explicit priority within UHC design and ongoing monitoring and evaluation plans. Care should also be taken not to embark on the path to UHC with undue haste and inadequate planning, or risk creating a scenario where the ‘inverse equity hypothesis’ [8] holds true (i.e. whereby interventions reach the most privileged groups first and then ‘trickle down’ to the poor and marginalised), effectively widening the disparity gap and undermining the true meaning and intent of UHC.

With UHC having been criticised for being too broad a goal, open to different interpretations, and disaccord around the meanings of its component parts (i.e., ‘universal’ , ‘access’ , ‘effective coverage’ and ‘need’) [13], it can be difficult for countries to best decide how to turn the idealistic goal of UHC into practical measures. This is particularly the case for low to middle income countries (LMICs) which are often following UHC models which have arisen in developed nations such as the United Kingdom and Japan. In practice, most countries tend to fall short of full universality - whether it be in the breadth (reaching all population groups), depth (the inclusion of all needed services) or height (the proportion of costs covered) [13]. While most countries aim for 100% breadth, they settle for depth that is either ‘limited’ (i.e. a minimum package of cost-effective interventions) or ‘strategic’ (i.e. scaling up of selected programmes which are especially important for disadvantaged groups such as MCH services) [13]. Too often, equity of access to effective coverage, comprised of utilisation, need and quality, is not prioritised or measured and as D’Ambruoso [14] points out, ‘incomplete analysis of equity can inadvertently maintain disadvantage and exclusion’. This paper thus sets out to assist policy and decision makers to understand how to prioritise and measure equity within universal health care systems. It does this by elucidating how equity is currently contextualised and measured within UHC and describing useful tools and resources which will assist decision makers to successfully plan and implement universal health coverage programmes which inherently ensure equity for all.

### Methodology

The methodology for the study is a narrative review [15]. This was chosen in favour of a conventional systematic review for its strength in constructing a critical analysis of a complex body of predominantly qualitative literature [16] and for allowing the delineation of equitable achievement of UHC to emerge from our analysis of the literature rather than being defined *a priori* [17]. The goal of the review was to identify seminal and empirical literature on the conceptual issues, theoretical debates and empirical evidence around the measurement and attainment of equity within universal health coverage programmes.

The authors retrieved peer-reviewed scientific literature published in English between 2005 and 2013 from PubMed database using the keywords ‘universal health coverage’ OR ‘universal health care’ OR ‘UHC’ combined with the derivations of the terms ‘equity’ OR ‘inequity’ (i.e. equity/equitable/equitably/equities/equitability, with the same derivations for inequity). These search terms however retrieved an unwieldy number of approximately 9,000 papers and hence the search terms were limited to the above-mentioned UHC derivations plus the simplified terms ‘equity’ and ‘inequity’. This search returned 66 results of which the titles and abstracts were reviewed to determine relevance to the research objectives. Papers were excluded if they did not focus on equity (of access, financial risk protection or health outcomes) within UHC in addition to focusing on one or more of the following: (i) the impact of UHC programmes, policies or interventions on equity (ii) describe indicators, measurement, monitoring and/or evaluation of equity within UHC, or (iii) describe tools or resources to assist with such measurement. One article which was identified for inclusion based on the title and abstract was excluded because it was not available from either the scholarly databases or publisher. A total of 18 peer-reviewed articles were included. Additionally, as similar studies [18,19] have shown that for reviews of complex evidence, formal protocol driven search strategies may fail to identify important resources, the authors also used the informal approach of referencing chaining to ensure that secondary research and non-research articles of theoretical importance to the topic were included. Accordingly, the following organisational websites were also searched for grey literature: WHO, World Bank (WB), UHC Forward and the Rockefeller Foundation. A purposive search of authors who have a prominent publishing record in equity (for example Gwatkin, Whitehead and Sengupta) was also conducted. The result of these informal mechanisms was the inclusion of an addition 13 items of grey literature including country case studies, reports from working groups and expert meetings, advocacy pieces and measurement tools/resources.

A preliminary review of the literature revealed a lack of empirically based publications. As such, the evidence-grading tools normally applied to a systematic review were not employed as they have been previously shown to exclude important expert information and pertinent empirical experience from published articles of a more conceptual nature [20]. Key details from the included papers were collated via a data extraction form adapted for the narrative review design which facilitated a simple summary archive of the bibliographic details of each resource, the study type, setting, findings and recommendations (See Additional file 1: Table S1). Using the data extraction form, thematic analysis was conducted to identify dominant themes which are relevant to the study objectives and which inform a narrative discourse around the evolution of the measurement and attainment of equitable UHC, as presented in the next section.

## Results

A large number of papers did not evaluate/measure equity as an outcome of UHC, but rather described it as an integral component of UHC and were thus excluded. Most of the peer-reviewed journal articles consisted of secondary data analysis of medical and administrative records (n = 10) and reviews or commentaries (n = 5). There was also one prospective longitudinal cohort study, one systematic review and one narrative review. The organizational reports consisted of several analytical frameworks for monitoring of UHC at country and global levels, meeting reports describing the discussion of relevant indicators and the conceptualisation of equitable UHC, and several tools for estimating lives saved and economic outcomes of eliminating in-country disparities. Of the papers which did formally evaluate equity within UHC, roughly even proportions were from high-income and low to middle income countries (LMICs). There were several publications from Canada [21-24] and one each from Australia [25] and Taiwan [26]. Papers from LMICs included several from Thailand [12,27,28], Mexico [29], Chile [30] and Brazil [8,31]. Studies from high-income countries tended to focus on access to specialised services and procedures such as mental health services [22,23], and procedures for circulatory disease [22,25]. They also focused on distinct populations such as children [23], the elderly [24] and psychiatric patients [22] rather than the population as a whole. Two studies showed that despite systems of free universal coverage, there was greater inequity, measured in terms of waiting times and receipt of procedures, for interventions which were non-urgent or elective, or for which there was a lack of clearly defined treatment protocols [21,25]. This reveals a need for further research to determine whether the higher rates of procedures for discretionary care are due to overuse in advantaged individuals or underuse in disadvantaged groups; both having distinctly different policy implications for high-income UHC settings.

In general, studies from LMICs did explore the impact of equitable UHC on access to a basic package of essential services and health outcomes for the entire population, most commonly disaggregated by geographical area, socio-economic status and gender. A finding which was fairly consistent across both developing and developed contexts was that a key area in which inequity may arise within UHC is through disparities in quality of care and access to specialised clinical services. For example, although Thailand witnessed an increase in the access and coverage of primary care following the 2001 introduction of national health insurance, closer inspection of data revealed a disparity in the type of health facilities being accessed by different socioeconomic groups [12]. While the rich received most of their health care through provincial/general hospitals and private clinics, the poor generally received care from the lowest level facilities, health centres. In effect the poor had less choice of service provider, inadequate referrals and hence a potentially restricted package of benefits. This finding was congruent with a systematic review conducted by Hanratty and colleagues which also documented a pro-rich bias in the use of curative specialist hospital services but reasonably equitable access to primary health care [10]. The authors concluded that further research focusing on how to more effectively measure and monitor equity in universal health systems, with particular attention on how to define “need” and measure quality is necessary.

The literature also reveals consensus on the fact that measurement and monitoring of UHC, and equity as an implicit component, remains challenging and is an evolving concept. The progressive conceptualisation and means of measuring equitable UHC are described further in several items of grey literature from key global health stakeholders. For example, the 2013 World Health Report: research for universal health coverage [32] describes UHC as complex and advocates for further research into how quality and equity of access are monitored within UHC. Accepting that the social determinants of health influence the equity of coverage, WHO urges countries to measure UHC via a spectrum ranging from inputs and processes, to outputs, outcomes and impact and that rather than trying to measure the coverage of all national health services, countries should choose a subset of services and associated indicators that are representative of the overall quantity, quality, equity and financing of services, disaggregated by locally appropriate dimensions (i.e. key socio-economic variables such as income, occupation, disability, etc.). Frenz and Vega’s [13] background paper for the 1st Global Symposium on Health Systems Research, titled, ‘Universal health coverage with equity: what we know, don’t know and need to know’ also argues that UHC policies must be measured by the effect they have on equity of access across the social gradient. They describe equity of access as ‘the just distribution of health care according to need’. In a review of the literature they however conclude that very few (n = 12) papers explicitly refer to equity of access relative to UHC goals, and that most research focuses on horizontal equity using equity of utilisation as a proxy indicator for equity of access. This substitution has however driven the research to focus on services and interventions for which there is readily available data and fails to adequately define or address unmet need for the most marginalised and disadvantaged populations who, for a variety of reasons, do not utilise the formal health system. The authors present an analytical framework for assessing equity of access in UHC policies which is based upon access being viewed as a multidimensional, multi-step process influenced by both supply and demand side factors. Within this framework, equitable access is seen as the experiences and interactions of different socioeconomic groups with the health care system, within the broader context of the structural inequities that define social hierarchies and hence determine differential health needs.

The 2008 World Health Report [11] identified raising the visibility of health inequities in public awareness and policy debates as a key mechanism to address health equity within primary health care. The resource, ‘Universal health coverage: a commitment to close the gap’ produced by the Rockefeller Foundation in collaboration with Save the Children, UNICEF and WHO [3] serves to do this within the UHC context. As evidenced by the title, this resource represents an advocacy tool – effectively displaying a highly visible commitment by key global health players to reduce health disparities within UHC. This report presents lessons learned from countries undergoing UHC reforms as well as practical tools to assist countries to prioritise efforts to close the gap in health. The ‘Lives Saved Tool’ (LiST) can be used to estimate the impact of eliminating in-country wealth inequities in coverage of MCH services and an econometric analysis tool estimates the impact of more equitable health financing on mortality rates. Additionally, the resource also identifies policy options that governments and donors should consider when implementing reforms for UHC and estimates the effect this could have on health outcomes, setting out the implications for the SDGs.

In a review of the impact of universal coverage schemes in the developing world, the World Bank [33] concludes that a focus on affordability alone is insufficient for improving access and advocates for a more holistic approach to the dimensions of access which must be explicitly incorporated into the design of UHC programmes. The paper by Jacobs and colleagues [34] is instructive in this area. The authors first analyse all of the monetary and non-monetary barriers to access reported in the literature and classifies them as either demand or supply side barriers. It then describes established interventions that could be implemented in low-income Asian countries at district level by the health sector alone or in collaboration with other government departments, nongovernment or civil-society organizations and through the public and/or private sectors. An analytical framework mapping the identified barriers and interventions against four dimensions of access (geographical access, availability, affordability and acceptability), is then created and applied to two case studies to demonstrate its utility in assisting policy makers and health planners to identify barriers, devise interventions and assess their appropriateness.

Further work to establish useful and comparable indicators to measure UHC and equity has been the focus of several recent high-level meetings. For example, the Health Systems 20/20 meeting in July 2012 focused on ‘Measuring and monitoring country progress towards universal health coverage: concepts, indicators, and experiences’ [35]. At this meeting it was agreed that the creation of a conceptual framework for UHC which uses ‘equity-catalysing’ indicators to measure financial risk protection (FRP) and coverage with good quality health services for all was a priority. The Bellagio Centre of the Rockefeller Foundation responded in September 2012 publishing a report titled, ‘Measurement of trends and equity in coverage of health interventions in the context of universal health coverage’ [36], presenting a first draft framework and criteria for the development of an index and tracer indicators for global monitoring. It ascertains that to progress towards UHC, regular measurement of equity is paramount and describes the types of information and indicators needed to monitor the key components of UHC namely; coverage, effective coverage and quality of care, financial hardship, and equity*.* The report advocates that the absolute performance of the disadvantaged is most critical within UHC and that trends in the least performing groups should receive at least as much, if not more attention, than the whole population (i.e. the progress of the most disadvantaged groups should be compared against pre-intervention levels as well as to relative measures against the most advantaged groups and to pre-determined targets).

This concept of UHC monitoring was further expanded on in the WHO technical meeting in September 2013 as described in the summary report, ‘Measurement and monitoring of universal health coverage’ [37]. Discussion around the selection of appropriate indicators for equity analyses saw participants agree that in addition to disaggregation by sex and age group, disaggregation by household wealth and geography (both urban/rural and subnational administrative levels) is essential for countries to monitor internal disparities. Ultimately however, it was seen as the remit of countries to select their own locally appropriate dimensions of inequity and the global research community must create internationally comparable, tracer indicators. More recently, in December 2013, the World Bank Group and WHO published an instructive resource, titled ‘Monitoring Progress towards Universal Health Coverage at Country and Global Levels: A Framework’ [38]. This framework sets out likely timelines for UHC achievement aligned with the proposed 2015–2030 focus of the SDG agenda. It is suggested that global monitoring focus on essential health services coverage including a set of interventions related to the MDGs (focusing on communicable diseases, reproductive health, and nutrition for mothers and children) and a set of interventions related to chronic conditions and injuries (CCIs), (addressing NCDs, mental health, and injuries across the life course). Financial risk protection would also be monitored based on the incidence of catastrophic health expenditures and impoverishment due to out-of-pocket health payments. Importantly, the framework proposes explicit ‘equity goals’ comparing the progress of the poorest 40% of the population against aggregate population levels for the indicators around service coverage and protection from catastrophic payment goals. As impoverishment due to health expenditure is considered of equal importance across all economic groups, only aggregate levels would be measured. It is the mandate of countries to decide on the appropriate indicators to measure their own burden of disease within their own specific context. While the framework encourages truly universal (i.e. 100%) coverage of essential health services, a more realistic ‘80:40’ target is proposed to ensure that the poorest 40% of the population receives at least 80% coverage for interventions addressing the MDGs and the CCIs. In terms of financial protection, 100% of the population should be protected from both impoverishing and catastrophic health payments. The report presents an illustration of how these targets could be applied using data from world health surveys which effectively confirms that few developing regions currently achieve this 80:40 target for coverage of CCIs, MDGs and FRP. Feedback is currently being sought on this proposed framework and its acceptance as an umbrella goal for the SDGs remains to be seen.

## Discussion

The literature shows that the measurement of universal health coverage and equity, although complex and in a somewhat conceptual stage, has become more definitive of late. It gives rise to a number of common themes which will be discussed below in the context of other research on preventing health inequity across the health system (i.e. not exclusively in UHC programmes).

The current study found that the majority of papers, which, by and large, consist of lessons learned in individual country case studies, were consistent in their findings that UHC programmes should focus first on increasing coverage and decreasing economic barriers to access amongst the most disadvantaged groups. This fits with the term, ‘*progressive universalism*’ which has more recently been coined by Gwatkin and Ergo [9], describing affirmative action strategically targeted at the most disadvantaged in the planning of UHC programmes. The authors describe the success of this approach in reducing inequality in coverage in two country examples: Brazil’s Family Health Programme and Mexico’s Popular Insurance initiative. Both of these programmes initially concentrated coverage amongst the most disadvantaged groups and then extended initiatives with declining subsidies to those with higher income levels. In Brazil this was done by first reaching deprived municipalities, whereas the Mexican programme used existing social security mechanisms to extend health insurance to those without cover. As such, the authors advocate that deliberate adoption and scaling up of strategies should be aimed at reaching the poorest first and that equity must be taken into account when assessing overall progress in coverage at country level by using stratified analyses. Victora et al. [39], similarly describe how by targeting MCH interventions at the poor and disadvantaged from the start of the programme, several ‘countdown to 2015’ countries circumvented the inverse equity hypothesis. Their analysis showed, for example, that in countries where additional focus was on targeting the poor, the uptake of insecticide treated bed-nets increased rapidly across *all* wealth quintiles. Conversely, in the absence of a pro-poor focus, the uptake of nets was slower across the whole population and congregated disproportionally in the rich. Additionally, inequality remained static in countries which had reductions or only minimal increases in coverage, highlighting the effectiveness of progressive universalism as an effective means of reducing inequity. It is however essential that countries have accurate mechanisms for determining which populations are poor or disadvantaged. In this respect, it is recommended that the ministry of health collaborate with key stakeholders such as the ministry of social welfare or donor and development partners involved in poverty reduction initiatives who may have pertinent experience in identifying and reaching such populations [40].

While the literature advocates for UHC interventions which increase financial risk protection, it is important for countries and global stakeholders to take a broad view of what equitable financing actually means. For example, Sengupta [41] questions what he describes as the ‘dominant universal insurance model’ in many LMICs which promotes public provision of high-demand primary care but privatisation of more profitable tertiary services. He claims that this approach weakens already fragile public health systems, and declares that the aforementioned UHC programmes in Mexico and Brazil, as well as those of Chile, Colombia, India and Thailand (which are usually based on pooling of funds through insurance and through increased private provision of services), have actually increased inequity by decreasing the efficiency of the publicly funded health system. For example, although the Mexican UHC scheme rapidly expanded insurance to a large portion of the population and led to reductions in catastrophic and impoverishing health expenditure [29], various insurers now provide different packages of benefits, resulting in fragmentation of the health system and associated reductions in efficiency [41]. The current review also revealed similar findings in the research describing inequities in receipt of specialised health care in Canada and Australia in spite of universal insurance systems. Furthermore, Sengupta criticises the growing private sector for drawing crucial health work-force and resources away from the public sector and weakening its capacity to provide quality services. He concludes that a single, publicly-funded health system is better placed to offer equitable health outcomes and is more affordable for LMICs as it limits market-driven price setting by private providers and insurance companies. It thus becomes apparent from both country case studies and expert commentary, that achieving equity within UHC requires a holistic approach focused on creating and strengthening networks of accessible and high quality primary, secondary and tertiary health care. However, systems that work to strengthen health systems across the continuum are more likely to reduce inequity in the long run and the design of UHC financing mechanisms should consider the equity implications for both individuals, key populations, and the health system itself.

The abovementioned resources have been included as useful references for countries as they plan UHC programmes and monitoring and evaluation frameworks for UHC. The articles provide an understanding of key principles for measurement of equity within UHC, documenting the evolution of a draft framework and indicators. It is important that countries assess the relevance of resources to their own context being mindful of the type of indicators and data they can reasonably collect and use within current resources. Most importantly, they should analyse the equity impact of their selected indicators in effectively protecting the most disadvantaged populations. For example, if countries are to adopt the newly-proposed ‘80:40’ targets created by the WHO and WB (which focus on wealth quintiles and do not explicitly cover other dimensions of inequity such as gender, race, disability etc.) this could actually serve to hide or even increase in-country disparities. As such, this proposed monitoring framework should be given careful consideration by both countries and global stakeholders.

## Conclusion

UHC has already located itself as a likely key to the transition from the MDGs to the post-2015 SDGs and must ensure that disadvantaged groups benefit as much as privileged ones by having an explicit focus on the measurement of equity. This review elucidates the changing position of equity within the UHC agenda, from being viewed as an integral component and implied outcome of UHC, to more recently being seen as a complex but measurable indicator of UHC success. This progressive contextualisation of UHC has led to a recent proliferation of tools and resources such as indicators and frameworks which aim to stimulate better definition and measurement of equity and UHC itself. Several lessons which have been learnt from countries implementing UHC regarding equitable access to a high quality range of health services provide valuable recommendations for other countries on the path to UHC. These resources and the discourse around their evolution will assist countries to consider their own requirements for monitoring and evaluating equity within their systems of universal health care and not falsely assume that equity is an inevitable outcome of UHC.

## Additional file

Additional file 1: Table S1. Summary of included papers and resources.
