# Supplementary material for: Achieving equity within universal health coverage: a narrative review of progress and resources for measuring success
Source: Int J Equity Health. 2014 Oct 10;13:72. doi: 10.1186/s12939-014-0072-8 (PMC4192297; doi:10.1186/s12939-014-0072-8)
Supplement: Additional file 1: Table S1. — Summary of included papers and resources. [file 12939_2014_72_MOESM1_ESM.docx]

Table 1 Summary of included papers and resources

| **Type of resource** | **Author details** | **Setting** | **Findings** | **Recommendations** |
| --- | --- | --- | --- | --- |
| Peer-reviewed journal article: a prospective longitudinal study | (Feigl and Ding, 2013) | Comparison of 194 countries | 75 countries have implemented UHC legislation however according to a derived index only 51 countries (26% of 194 countries) have achieved UHC. | Further research needs to be done to define achievement and measurement of UHC. |
| Peer-reviewed journal article: secondary data analysis using decomposition analysis of concentration indexes | (Yiengprugsawan et al., 2011) | Thailand following the implementation of national health insurance | Disparity in type of health facilities being accessed by SES; the better off tended to receive health care through hospitals and private clinics while the poor received most of their care at health centres. | Future policies need to focus on the quality of primary care and ensure equitable referrals to specialised care. |
| Peer-reviewed journal article: systematic review | (Hanratty et al., 2007) | Equity in the use of curative services in countries with established market economies with UHC. | Pro-rich bias in use of specialist hospital services and reasonably equitable access to primary health care by different SES groups. | Improvements are needed in the way that equity in universal systems is monitored, with particular attention to how "need" is defined and to the impact on patients of indirect costs. |
| Peer-reviewed journal article: commentary | (D'Ambruoso, 2013) | Global health post-2015 | Equity (as distinct from equality) does not feature prominently in the emerging post-2015 and SDG consultative frameworks. | *Universal health equity* should be an operational and analytical priority in the post-2015 development agenda. |
| Peer-reviewed journal article: comment | (Gwatkin and Ergo, 2011) | Global, UHC | UHC is difficult to achieve and an increasing volume of empirical evidence suggest that the ‘trickle-down effect’ is pervasive and may constitute the norm. | Countries should implement a progressive universalism approach to UHC, i.e. they should aim to first increase coverage among disadvantaged groups, rather than taking the traditional approach of serving initially those who are easiest to reach. |
| Peer-reviewed journal article: commentary | (Knaul et al., 2012) | Mexico | Mexico has made significant progress with social protection of health based on access to effective health care being viewed as a universal right. Research shows that the program is improving access to health services, improving effective coverage and reducing catastrophic and impoverishing health expenditure particularly for the poor. | Further reform including systematic measures to reorganise the health system by functions is needed to continue progress towards UHC. |
| Peer-reviewed journal article: an analytical framework | (Jacobs et al., 2011) | Low to middle income Asian countries | Presents an analytical framework for selecting appropriate interventions to address barriers (geographical access, availability, affordability and acceptability) to health services. The framework can be used both to identify interventions and to analyse why certain interventions fail to tackle specific barriers. | A combination of interventions concurrently addressing demand-side and supply-side barriers are required to tackle specific access barriers but their effectiveness can be influenced by contextual factors. |
| Peer-reviewed journal article: secondary analysis of cohort records | (Kisely et al., 2007) | Nova-scotia province of Canada | Despite the UHC system, psychiatric status affects survival with and access to some procedures for circulatory disease. Despite increased risk of death, psychiatric patients, and in particular, inpatients were less likely to undergo specialised or revascularization procedures. | Further research into how disparities arise and how to reduce them is a key priority for future research. |
| Peer-reviewed journal article: primary and secondary analysis | (Blais et al., 2003) | Canada – Quebec province | While no significant regional differences were found for need indicators, mental health services for children are not distributed across regions according to need. | More evidence-based planning is required, specifically using epidemiologic survey data, to match resources to needs and to monitor changes over time. |
| Peer-reviewed journal article: cross-sectional analysis of secondary data | (Cunningham et al., 2011) | Canada: British Columbia Province | Inequities were observed in end-of-life health care with populations with higher income receiving more care and need-adjusted equity results suggesting that males received less care than females. | Identifying potential systemic reasons for the observed patterns of inequity is important in designing policies to address such patterns. |
| Peer-reviewed journal article: retrospective cohort analysis of secondary data | (Korda et al., 2009) | Australia – 12,940 patients hospitalised for acute myocardial infarction or angina in the state of Western Australia. | Using the SEIFA index of socioeconomic disadvantage, the results showed Australia’s health care system does not guarantee equity in the receipt of high technology health care for patients with IHD. There was more equity in the receipt of procedures for AMI, where the guidelines for treatment are well established, but less equity for angina patients, with more advantaged populations receiving more procedures for angina, perhaps because health care is less urgent and more discretionary. | Further research is needed to determine whether the higher rates of procedures for discretionary care are due to overuse in advantaged individuals or underuse in disadvantaged groups – with different implications for policy. |
| Peer-reviewed journal article: cross-sectional study using a multi-criteria decision making approach with grey incidence analysis | (Kreng and Yang, 2011) | Taiwan under the national health insurance system and the 23 decision making units. | There was found to be horizontal inequity of resource allocation between geographic areas with the North receiving more resources with limited access to health care services in rural areas. | Under the NHI scheme payments for health care suppliers could be adopted as an efficient strategy to reduce the disparity of resource allocation and ensure more equitable distribution. Further emphasis should be put on vertical equity taking into account demographics (i.e. the elderly need more care) as well as the social environment (i.e. issues with transport in rural areas). |
| Peer-reviewed journal article: secondary analysis of medical records | (Shortt and Shaw, 2003) | Review of 39,000 surgeries performed between 1992 and 1999 at two Canadian hospitals. | A review was conducted of the waiting times for elective surgery by SES however no relationship was determined. | Further research needs to be done to include the population who did not receive elective surgery and to adjust for severity of illness. |
| Organisational report: The World Bank systematic review and synthesis of existing evidence | (Giedion et al., 2013) | The developing world | UHC interventions in LMICs improve access to health care. The evidence also shows, though less convincingly, that UHC often has a positive effect on financial protection, and that, in some cases it seems to have a positive impact on health status. | 1. Affordability is important but may not be enough.  2. Target the poor, but keep an eye on the non-poor.  3. Benefits should be closely linked to target populations’ needs.  4. Highly focused interventions can be a useful initial step toward UHC.  5. Incorporate evaluation in the early stages of the program and, ideally, simultaneously design the intervention and evaluation. |
| Organizational report: WHO World Health report 2013 | (World Health Organization, 2013) | Global, UHC | Proposes that countries measure UHC across a spectrum which encompasses the social determinants of health across a subset of services that represent overall quantity, quality, equity and financing of services, disaggregated by locally-appropriate dimensions. | Further research is needed into how quality and equity of access are monitored within UHC programmes. |
| Background paper for Global Symposium on Health Systems Research | (Frenz and Vega, 2010) | Literature review and recommendations | Equity of utilisation is being commonly used as a proxy indicator for equity of access but fails to adequately capture unmet need of those who do not use the formal health sector. Presents an analytical framework for assessing equity of access within UHC policies. | Equitable access should be viewed as the experiences and interactions of different groups with the health system, within the broader social determinants of health. |
| Organisational meeting report | (USAID, 2012) | Country-level | There are lingering conceptual challenges with measuring UHC and varying objectives and audiences for indicators: global advocacy purposes require a small number of simple, intuitive, easy-to-communicate indicators whilst country-level health planners and policymakers, need more comprehensive, detailed, and actionable country-specific indicators for policy and management. | There is a need for a conceptual framework to measure UHC utilising equity catalysing indicators which encompass financial risk protection and coverage of good quality health services for all. A meeting of country-level stakeholders should be convened to facilitate common understanding and identify country-level needs for indicators. Concurrently, key stakeholders should come to consensus on criteria for selecting high-level indicators. |
| Organization report: Rockefeller Foundation | (UHC Forward, 2012) | Global monitoring of health intervention coverage, with a focus on equity within UHC | Presents a draft framework and criteria for the development of an index and tracer indicators for global monitoring of UHC with regular measurement of equity. | The progress of the most disadvantaged/least performing groups should receive as much, if not more attention than the whole population. |
| Organization report: WHO technical meeting report | (World Health Organization et al., 2013) | Global and country-level UHC | Discusses appropriate indicators for equity analyses at global and country levels. | Countries must select their own indicators and global stakeholders should determine internationally comparable, tracer indicators for UHC. |
| Organization report: County and Global monitoring frameworks for UHC | (World Health Organization and World Bank, 2013) | Global and country-level UHC | Presents a framework and timelines for monitoring of UHC covering a set of interventions related to the MDGs, as well as related to chronic conditions and injuries, as well as financial risk protection. | Feedback is sought on the draft framework and its potential as an umbrella goal for the SDGs. |
| Organisational report: Occasional Paper | (Sengupta, 2013) | Global | Secure finances for health care are a necessary but insufficient condition for equitable and high quality care. Equity and efficiency tend to be compromised by ideological pressures which prevent an entirely public system of care provision. | The only to build truly universal health outcomes is for finances to be administered by well-designed public health care systems rather than the private sell-out of health systems via UHC. |
| Organisational report: structured review of country experiences and literature, key  informant interviews,  econometric analyses, a Lives Saved Tool analysis and case studies. | (Brearley et al., 2013) | Global | The report estimates the number of maternal and child health lives that would be saved if within-country health inequities in the coverage of essential MCH interventions were eliminated. | 1.) The level and progressivity of funding for the health sector must increase.  2.) Health sector resources must be pooled across the population  3.) The package of benefits should be tailored to meet the needs of poor and vulnerable people, including a minimum of free primary healthcare, and aligning the incentives of healthcare providers through payment mechanisms, will help to ensure more equitable coverage.  4.) Quality concerns in service delivery must be addressed.  5.) Coordinated reforms across the whole system, and beyond the health sector, are needed to address other barriers to demand and supply.  6.) The quantity, quality and use of disaggregated data is critical to inform planning process, monitoring, evaluation and accountability.  7.) Effective government stewardship is essential for regulation, strategic planning and effective collaboration with other actors. |

1. BLAIS, R., BRETON, J. J., FOURNIER, M., ST-GEORGES, M. & BERTHIAUME, C. 2003. Are mental health services for children distributed according to needs? *Can J Psychiatry,* 48**,** 176-86.
2. BREARLEY, L., MARTEN, R. & O'CONNELL, T. 2013. Universal Health Coverage: A Commitment to Close the Gap.
3. CUNNINGHAM, C. M., HANLEY, G. E. & MORGAN, S. G. 2011. Income inequities in end-of-life health care spending in British Columbia, Canada: A cross-sectional analysis, 2004-2006. *Int J Equity Health,* 10**,** 12.
4. D'AMBRUOSO, L. 2013. Global health post-2015: the case for universal health equity. *Glob Health Action,* 6**,** 19661.
5. FEIGL, A. B. & DING, E. L. 2013. Evidenced Formal Coverage Index and universal healthcare enactment: A prospective longitudinal study of economic, social, and political predictors of 194 countries. *Health Policy,* 113**,** 50-60.
6. FRENZ, P. & VEGA, J. 2010. Universal health coverage with equity: what we know, don't know and need to know. *Background paper for the global symposium on health systems research, 16-19 November 2010 - Montreux, Switzerland.* HSR Symposium.
7. GIEDION, U., ALFONSO, E. & DIAZ Y 2013. The impact of universal coverage schemes in the developing world: a review of the existing evidence. Washington DC,: The World Bank.
8. GWATKIN, D. R. & ERGO, A. 2011. Universal health coverage: friend or foe of health equity? *The Lancet,* 377**,** 2160-2161.
9. HANRATTY, B., ZHANG, T. & WHITEHEAD, M. 2007. How close have universal health systems come to achieving equity in use of curative services? A systematic review. *International Journal of Health Services,* 37**,** 89–109.
10. JACOBS, B., IR, P., BIGDELI, M., ANNEAR, P. L. & VAN DAMME, W. 2011. Addressing access barriers to health services: an analytical framework for selecting appropriate interventions in low-income Asian countries. *Health Policy and Planning,* 2011**,** 1-13.
11. KISELY, S., SMITH, M., LAWRENCE, D., COX, M., CAMPBELL, L. A. & MAATEN, S. 2007. Inequitable access for mentally ill patients to some medically necessary procedures. *CMAJ,* 176**,** 779-84.
12. KNAUL, F. M., GONZÁLEZ-PIER, E., GÓMEZ-DANTÉS, O., GARCÍA-JUNCO, D., ARREOLA-ORNELAS, H., BARRAZA-LLORÉNS, M., SANDOVAL, R., CABALLERO, F., HERNÁNDEZ-AVILA, M., JUAN, M., KERSHENOBICH, D., NIGENDA, G., RUELAS, E., SEPÚLVEDA, J., TAPIA, R., SOBERÓN, G., CHERTORIVSKI, S. & FRENK, J. 2012. The quest for universal health coverage: achieving social protection for all in Mexico. *The Lancet,* 380**,** 1259-79.
13. KORDA, R. J., CLEMENTS, M. S. & KELMAN, C. W. 2009. Universal health care no guarantee of equity: comparison of socioeconomic inequalities in the receipt of coronary procedures in patients with acute myocardial infarction and angina. *BMC Public Health,* 9**,** 460.
14. KRENG, V. B. & YANG, C. T. 2011. The equality of resource allocation in health care under the National Health Insurance System in Taiwan. *Health Policy,* 100**,** 203-10.
15. SENGUPTA, A. 2013. Universal Health Coverage: Beyond rhetoric. *In:* MCDONALD, D. A. & RUITERS, G. (eds.) *Municipal Services Project, Occasional Paper No. 20 - November 2013.*
16. SHORTT, S. E. & SHAW, R. A. 2003. Equity in Canadian health care: does socioeconomic status affect waiting times for elective surgery? *CMAJ,* 168**,** 413-6.
17. UHC FORWARD 2012. Measurement of trends and equity in coverage of health interventions in the context of universal health coverage. Rockefeller Foundation Center, Bellagio, September 17-21, 2012.
18. USAID 2012. Measuring and monitoring country progress towards universal health coverage: concepts, indicators, and experiences. Meeting summary - July 20, 2012, Washington, DC. Washington DC: United States Agency for International Development, Health Systems 20/20.
19. WORLD HEALTH ORGANIZATION 2013. The World Health Report 2013: research for universal health coverage. Geneva: WHO.
20. WORLD HEALTH ORGANIZATION & WORLD BANK 2013. Monitoring Progress towards Universal Health Coverage at Country and Global Levels: A Framework. *Joint WHO/World Bank Group Discussion Paper, December 2013*
21. WORLD HEALTH ORGANIZATION, WORLD BANK & MINISTRY OF HEALTH SINGAPORE 2013. Measurement and monitoring of universal health coverage. Techinical meeting; Singapore, 17-18 September 2013 summary report.
22. YIENGPRUGSAWAN, V., CARMICHAEL, G., LIM, L.-Y., SEUBSMAN, S. & SLEIGH, A. 2011. Explanation of inequity in utilization of ambulatory care before and after universal health insurance in Thailand. *Health Policy and Planning,,* 26**,** 105-114.
